# Case report: Tumor collision in the colon, adenocarcinoma – lymphoma

**DOI:** 10.1016/j.ijscr.2022.107573

**Published:** 2022-08-31

**Authors:** Michael Zapata Palomino, Isabella Caicedo-Holguín, Stephania Pardo, Andrea Tovar Mera, Alden Pool Gómez, José Omar Zorrilla

**Affiliations:** a Department of General Surgery, Universidad del Valle, Hospital Universitario del Valle, Cali, Colombia; b Lili Valley Foundation, Clinical Research Center, Cra 98 No. 18 - 49, Cali 760032, Colombia; c School of Medicine, Universidad del Valle, Hospital Universitario del Valle, Cali, Colombia; d Department of Surgical Oncology, Universidad del Valle, Hospital Universitario del Valle, Cali, Colombia

**Keywords:** Tumor collision, Colon adenocarcinoma, Colon lymphoma, Case report, HIV

## Abstract

**Introduction:**

Tumor collision is the cohesion in a time of 2 tumors in the same space or organ but of different cell lineage.

**Presentation of case:**

72-year-old patient with a history of black stools, involuntary weight loss and moderate protein-calorie malnutrition, asthenia, and adynamia; with endoscopic studies with the finding of esophageal candidiasis that leads to taking a rapid test for HIV (human immunodeficiency virus) which is positive; CT (computerized axial tomography) of the abdomen is performed, showing thickening of the cecum and distal ileum, as well as striation of fat in the same area, findings related to a primary neoformative process, for which it is decided to carry out a right hemicolectomy laparoscopically with pathology that reports ulcerated moderately differentiated adenocarcinoma that invades up to the muscle layer, associated with lymphoid proliferation with immunohistochemical findings consistent with lymphoplasmablastic lymphoma, this is probably linked to his diagnosis of HIV, configuring the diagnosis of tumor collision; extension studies with no findings of metastatic neoplastic disease.

**Discussion:**

Tumor collision is a rare entity, which implies failure in the genomic control and replication sites of different cell lines, which, due to their lineage, have different regulatory processes, all this occurring at the same time in the same tissue.

**Conclusion:**

The casuistry for collision tumors is scarce; even in the reference centers for oncology, the treatment is challenging given the multiple variables to consider, the particularity of each case, and the scarce evidence on the subject.

## Highlights

• Tumor collision is the cohesion of two different tumors in the same space.
• It is a rare entity often diagnosed in surgery or by the pathologist
• Collision tumors pose a challenge in determining the prognosis of the patient
• As collision tumors are composed of two different tumors, the recommended treatment is to target the most aggressive neoplasm.

## 1 Introduction

Tumor collision is the cohesion in a time of two tumors in the same space or organ but of different cell lineage [1], [2], [3], [4], [5], [6], [7]. The cause of this phenomenon is not yet understood, there are multiple case reports and some case series, but due to its low incidence and heterogeneity in presentations, each case is challenging [1], [2]. Additionally, the scarce consensus in the literature and its histopathological variation makes it difficult to determine the management and prognosis of these lesions [1].

The existing bibliography focuses on cases of tumors originating in skin cells, squamous cells, melanoma, spindle cell, and gastrointestinal tumors of the upper digestive tract, with emphasis on the stomach [4], [8], [9], [10], [11]. However, there are few reports of collision tumors in the colon and even less with the variant of colonic adenocarcinoma in collision with lymphoma [1], [2]. This case report was made according to the checklist of SCARE guidelines. [12]

## 2 Presentation of case

She is a 72-year-old female patient who goes to the emergency department due to a history of black stools, involuntary loss of sixteen kilograms of weight in the last six months, asthenia, and adynamia. The physical examination and admission laboratories documented severe normochromic normocytic anemia and moderate malnutrition. An upper digestive tract endoscopy showed esophageal candidiasis Kodsi grade I, (Table 1), and the colonoscopy reported a mass in the ascending colon with a macroscopic description of moderately differentiated adenocarcinoma. [13]

**Table 1:** Kodsi classification.

Kodsi classification for endoscopy severity of esophageal candidiasis	
Grade I	Few raised lesions (<2 mm) without surrounding edema nor laceration
Grade II	Multiple raised lesions (>2 mm) without surrounding edema nor laceration
Grade III	Linear, nodular and confluent lesions
Grade IV	Same as grade III with narrowing of the lumen and friability of the mucosa
Grade V	Thick white plaque covering the lumen in circumferential manner causing narrowing of the lumen
Grade VI	Endoscopy can detect oropharyngeal candidiasis

The oncology surgery department performed staging tests, including abdominal tomography, that showed thickening of the cecum and distal ileum and fat striation in the same area. Additionally, given the endoscopic finding of candidiasis, a rapid HIV test was performed, which was positive; the viral load was >1,000,000 copies and an LTCD4 count <50. The patient begins nutritional recovery and follow-up by infectology.

Posteriorly started antiretroviral therapy (ART) and preoperative optimization; 20 days later, she underwent laparoscopic right hemicolectomy with side-to-side ileocolic anastomosis with a mechanical stapler. After the surgical procedure, she presented good evolution, tolerance to oral feeding, pain modulation, and normal stools, which is why she was discharged on the fifth postoperative day with ambulatory evaluations by a multidisciplinary team.

**Fig. 1:**
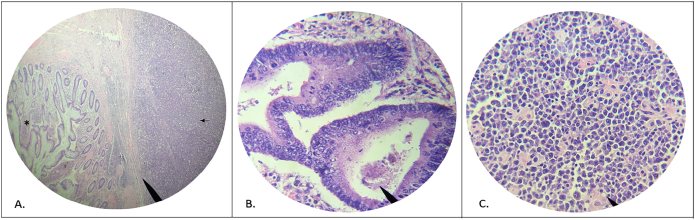
A. Low magnification photomicrograph (40×) showing tumor collision: (*) Moderately differentiated adenocarcinoma of the colon, (←) Lymphoplasmablastic lymphoma. B. Adenocarcinoma at higher magnification (100×). Lymphoplasmablastic lymphoma at higher magnification (100×).

In the following ambulatory control, the post-surgical pathology reports ulcerated moderately differentiated adenocarcinoma, tumoral size of 8 × 6 × 3.2 cm which invades the muscle layer associated with a hematolymphoid proliferation to be classified with immunohistochemical studies, Fig. 1. Linfovascular and perineural invasion not identified, resection border radial, proximal, and distal without disease, 0/32 ganglions which compromise the cecum and the cecal appendix. The TNM Classification was T2N0M0.

**Fig. 2:**
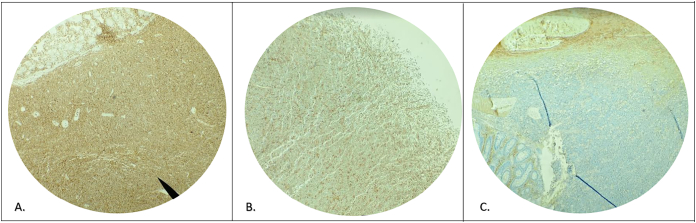
Immunohistochemical characterization of lymphoplasmablastic lymphoma, with positive staining for CD38 (A) and restriction of Kappa light chains (B) vs. Lambda (C).

The immunohistochemistry report shows tumor cells with positivity for CD45, CD138 and CD38 were evidenced in a diffuse, intense way and in a weak and heterogeneous way for CD30. Some helpers T lymphocytes were positive for CD3, CD2, CD5, and CD7. Kappa chain restriction and a Ki67 cell proliferation index of 95 % were observed. Additionally, there was no evidence of microsatellite instability or alteration in MLH1, MSH2, MSH6, and PMS2, configuring the diagnosis of tumor collision. Fig. 2.

The patient is currently undergoing follow-up and treatment by hematology and oncology, who defined no indication of adjuvant therapy at the moment for adenocarcinoma but started treatment for lymphoma with CHOP therapy (Cyclophosphamide, Doxorubicin, Vincristine, and prednisone).

## 3 Discussion

Tumor collision is a rare entity that involves a failure in the genomic control and replication sites of different cell lines, which have different regulatory processes due to their lineage. It is defined as the existence of two histologically distinct tumors, adjacent without a mixture or intermediate cell population, present in the same organ [1], [2], [3], [4], [5], [6]. Due to these characteristics, its approach, prognosis, and adjuvant therapy are challenging for the treating group. Additionally, the follow-up and the indications for a new surgical intervention are difficult.

Adenocarcinoma is the most frequent malignant tumor in the colon. However, it is unusual and has a few reported cases in the literature when it collides with another cell line and even lesser when it occurs with a lymphoma. [1], [3]. This association tends to be more frequent at the gastric level and is associated with neuroendocrine and carcinoid tumors, among others.

Different theories have described the origin of this phenomenon, including the cancerization theory, which describes a process in which an exposure factor affects a particular body region, developing two separate neoplasms in the same place [4]. The interaction theory indicates neoplasia that induces changes in the tissue generating second neoplasia [4]. Theories of aberration of immunohistochemical markers, where two neoplasms originate from a precursor stem cell, and one that explains that both tumors develop by colliding due to random reasons [1], [2], [3], [4]. However, there is still no explanation that satisfies all the possible associations of colliding cell lines.

In this case, the patient was predisposed to both types of tumors. Age has a predominant role in the genesis of replication errors making possible the development of an adenocarcinoma tumor. Additionally, due to its severe immunosuppression, lymphoid-type neoplasms are present to a greater degree and with greater severity. This case highlights the importance of a complete analysis of the extracted surgical piece, where the pathology group must be familiarized with these unusual but relevant findings for the adequate treatment of the patient.

The greatest challenge for the treating group is to determine if the pathological stage found in the surgical piece underestimates or overestimates the prognosis [1], [4]. They are given by the intrinsic characteristics of each tumor and their different response to each class of systemic treatments offered. Additionally, they may differ in the possibility of future surgical interventions, whether curative or palliative. Bulte et al., in a review of the literature on tumor collision in dermatological pathology, propose that these cases should be managed according to the scheme known for the most aggressive lesion and determine the prognosis according to this as well [4]. Although this seems to be an adequate strategy for managing these patients, there is not enough evidence to determine the treatment of tumor collision in the colon. In this case, it was decided to start CHOP therapy, given the aggressiveness of the lymphoid tumor.

## 4 Conclusion

The casuistry for collision tumors is limited. Even in reference centers for oncology management, treatment is challenging due to the multiple variables to consider, the particularity of each case, and the insufficient evidence. In addition, the prognosis is uncertain because there is no dual classification system for the different tumor responses to surgical and oncospecific management. However, the approach, treatment, and follow-up must be carried out by a multidisciplinary team of the oncology board that allows the best decisions to be made in the complex therapeutic process of this sad coincidence of risk factors, genetic predisposition, and cell mutations.

## Abbreviations

HIV: human immunodeficiency virus
CT: computerized axial tomography
CD45: cluster of differentiation 45
CD138: cluster of differentiation 138
CD38: cluster of differentiation 38
CD30: cluster of differentiation 30
CD3: cluster of differentiation 3
CD2: cluster of differentiation 2
CD5: cluster of differentiation 5
CD7: cluster of differentiation 7
MLH1: MutL Homology, mismatch repair system component
MSH2: MutS Homolog 2
MSH6: MutS Homolog 6
PMS2: PMS1 Homolog 2, mismatch repair system component
LTCD4: lymphocytes positives for the cluster of differentiation 4
TARV: antiretroviral therapy
CHOP: Cyclophosphamide, Doxorubicin, Vincristine, and prednisone
Ki67: index of cellular proliferation

## Consent

Written informed consent was obtained from the patient and his family members to publish this case report and accompanying images. On request, a copy of the written consent is available for review by the Editor-in-Chief of this journal.

## Provenance and peer review

Not commissioned, externally peer-reviewed.

## Ethical approval

The study is exempt from ethical approval in our institution.

## Funding

The authors declared no funding for this research.

## Guarantor

Michael Zapata Palomino.

## Research registration number

Not applicable.

## CRediT authorship contribution statement

Study conception and design: Michael Zapata Palomino, Andrea Tovar Mera. Acquisition of data: Michael Zapata Palomino, Isabella Caicedo Holguín, Stephania Pardo. Analysis and interpretation of data: Michael Zapata Palomino, Isabella Caicedo Holguín. Drafting of manuscript: Michael Zapata Palomino. Isabella Caicedo Holguin. Stephanie Pardo. Critical review: Alden Pool Gómez, José Omar Zorrilla. All authors read and approved the final manuscript to submit.

## Declaration of competing interest

The authors declared no conflict of interest.
